# Supplementary material for: Cohort profile: population-based cohorts of patients with colorectal cancer and of their relatives in Geneva, Switzerland
Source: BMJ Open. 2022 Aug 25;12(8):e063914. doi: 10.1136/bmjopen-2022-063914 (PMC9422819; doi:10.1136/bmjopen-2022-063914)
Supplement: Author's manuscript [file bmjopen-2022-063914.draft_revisions.pdf]

BMJ Open is committed to open peer review. As part of this commitment we make the peer review history of every article we publish publicly available.

When an article is published we post the peer reviewers' comments and the authors' responses online. We also post the versions of the paper that were used during peer review. These are the versions that the peer review comments apply to.

The versions of the paper that follow are the versions that were submitted during the peer review process. They are not the versions of record or the final published versions. They should not be cited or distributed as the published version of this manuscript.

BMJ Open is an open access journal and the full, final, typeset and author-corrected version of record of the manuscript is available on our site with no access controls, subscription charges or pay-per-view fees (http://bmjopen.bmj.com).

If you have any questions on BMJ Open's open peer review process please email info.bmjopen@bmj.com

BMJ Open

Cohort profile: A population-based cohort of colorectal cancer patients in Geneva, Switzerland

Journal:	*BMJ Open*
Manuscript ID	bmjopen-2022-063914
Article Type:	Cohort profile
Date Submitted by the Author:	21-Apr-2022
Complete List of Authors:	Benhamou, Simone; University of Geneva, Global Health Institute, Geneva Cancer Registry; Research Centre on Epidemiology and Population Health, INSERM Unit 1018<br>Fournier, Evelyne; University of Geneva, Global Health Institute, Geneva Cancer Registry<br>Puppa, Giacomo; Geneva University Hospitals, Department of Clinical Pathology<br>McKee, Thomas; Geneva University Hospitals, Department of Clinical Pathology<br>Ris, Frédéric; Geneva University Hospitals, Division of Digestive Surgery<br>Rubbia-Brandt, Laura; Geneva University Hospitals, Department of Clinical Pathology<br>Viassolo, Valeria; Geneva University Hospitals, Oncogenetics Unit, Division of Oncology; Geneva University Hospitals, Division of Genetic Medicine<br>Zilli, Thomas; Geneva University Hospitals, Radiation Oncology; University of Geneva, Faculty of Medicine<br>Zlobec, Inti; University of Bern, Institute of Pathology<br>Chappuis, Pierre; Geneva University Hospitals, Oncogenetics Unit, Division of Oncology; Geneva University Hospitals, Division of Genetic Medicine<br>Rapiti, Elisabetta; University of Geneva, Geneva Cancer Registry, Global Health Institute
Keywords:	EPIDEMIOLOGY, Gastrointestinal tumours < GASTROENTEROLOGY, PUBLIC HEALTH

SCHOLARONE™
Manuscripts

I, the Submitting Author has the right to grant and does grant on behalf of all authors of the Work (as defined in the below author licence), an exclusive licence and/or a non-exclusive licence for contributions from authors who are: i) UK Crown employees; ii) where BMJ has agreed a CC-BY licence shall apply, and/or iii) in accordance with the terms applicable for US Federal Government officers or employees acting as part of their official duties; on a worldwide, perpetual, irrevocable, royalty-free basis to BMJ Publishing Group Ltd ("BMJ") its licensees and where the relevant Journal is co-owned by BMJ to the co-owners of the Journal, to publish the Work in this journal and any other BMJ products and to exploit all rights, as set out in our licence.

The Submitting Author accepts and understands that any supply made under these terms is made by BMJ to the Submitting Author unless you are acting as an employee on behalf of your employer or a postgraduate student of an affiliated institution which is paying any applicable article publishing charge ("APC") for Open Access articles. Where the Submitting Author wishes to make the Work available on an Open Access basis (and intends to pay the relevant APC), the terms of reuse of such Open Access shall be governed by a Creative Commons licence – details of these licences and which Creative Commons licence will apply to this Work are set out in our licence referred to above.

Other than as permitted in any relevant BMJ Author's Self Archiving Policies, I confirm this Work has not been accepted for publication elsewhere, is not being considered for publication elsewhere and does not duplicate material already published. I confirm all authors consent to publication of this Work and authorise the granting of this licence.

For peer review only

Cohort profile: A population-based cohort of colorectal cancer patients in Geneva, Switzerland

Simone Benhamou [1,2*], Evelyne Fournier[1], Giacomo Puppa[3], Thomas McKee[3], Frédéric Ris[4], Laura Rubbia-Brandt[3], Valeria Viassolo[5,6], Thomas Zilli[7], Inti Zlobec[8], Pierre Olivier Chappuis[5,6], Elisabetta Rapiti[1]

[1] Geneva Cancer Registry, Global Health Institute, University of Geneva, Geneva, Switzerland

[2] Research Centre in Epidemiology and Population Health, INSERM Unit 1018, Villejuif, France

[3.]Department of Clinical Pathology, Geneva University Hospital, Geneva, Switzerland

[4] Division of Digestive Surgery, University Hospitals of Geneva, Genève 1211, Switzerland

[5] Oncogenetics Unit, Division of Oncology, Geneva University Hospitals, Geneva, Switzerland

[6] Division of Genetic Medicine, Geneva University Hospitals, Geneva, Switzerland

[7] Radiation Oncology, Geneva University Hospital, Geneva, Switzerland, and Faculty of Medicine, Geneva University, Geneva, Switzerland

[8] Institute of Pathology, University of Bern, Murtenstrasse 31, 3008, Switzerland

*corresponding author

Professor Simone Benhamou

Geneva Cancer Registry,

Global Health Institute,

University of Geneva, Geneva, Switzerland

simone.benhamou@inserm.fr

For peer review only - http://bmjopen.bmj.com/site/about/guidelines.xhtml

ABSTRACT

Purpose: Colorectal cancer is the 3rd leading cause of cancer death worldwide. Variability between patients in prognosis and treatment response is partially explained by traditional clinico-pathological factors. We established a large population-based cohort of colorectal cancer patients and their first- and second-degree relatives registered in the Canton of Geneva, to evaluate the role of family history and tumor biomarkers on patient outcomes and improve systematic treatment selection and risk stratification.

Participants: The cohort population includes all colorectal cancer patients diagnosed between 1985 and 2013. Detailed information on patient and tumor characteristics, treatment and outcomes were extracted from the Geneva Cancer Registry database, completed by medical records review, and linkage with administrative and oncogenetics databases. Next-generation tissue microarrays (ngTMA) were constructed for approximately 70% of the cohort patients from formalin-fixed paraffin-embedded tissue samples of the primary tumor. A prospective follow-up of the cohort is realized annually to collect data on recurrences, second cancer and survival.

Findings to date: The cohort includes 5499 patients (4244 colon cancer patients and 1255 rectal cancer patients). Only 3% of patients were diagnosed during a screening procedure. Thus, the great majority of patients were diagnosed because of occurrence of symptoms and almost half of cases were diagnosed with an advanced disease. At the end of 2019, 337 local recurrences, 1143 distant recurrences, and 4035 deaths were reported.

Future plans: The cohort will be used for long-term studies of colorectal cancer epidemiology with focus on clinico-pathological factors and molecular markers. These data will be correlated with the most up-to-date follow-up data.

For peer review only - http://bmjopen.bmj.com/site/about/guidelines.xhtml

STRENGTHS AND LIMITATIONS

- This study, using a powerful resource based on regional record-linkage analysis of routinely collected data, will allow having one of the most comprehensive cohorts of CRC patients and their relatives, at a population level.

- Considerable insights into the natural history of CRC and the mechanisms through which epidemiological, clinicopathological, and molecular markers may alter the prognosis are expected. This knowledge will provide evidence to guide health professionals in the stratification and management of patients with this disease, and surveillance of their relatives.
- Weaknesses of the cohort are missing data for some of the recorded variables and the absence of tumor samples for approximately 30% of CRC patients.

For peer review only

INTRODUCTION

In Europe, colorectal cancer (CRC) is the second most common malignancy in women and the third one in men [1]. In 2020, 281,714 men and 238,106 women were diagnosed with CRC, and it accounted for approximately 13% of all cancer-related deaths in both genders. More than 80% of all CRC occur at the age of 60 years or more.

In high-income countries, the risk of CRC has been ubiquitously associated with environmental factors, such as dietary patterns (diet rich in fat and meat and poor in unrefined cereals and fiber), obesity, and sedentary lifestyle [2].

Approximately 6% of patients with CRC carry germline mutations in genes responsible of inherited genetic predisposition to CRC [3]). Lynch syndrome is the most common hereditary CRC syndrome and has been implicated in 2% to 5% of newly diagnosed CRC cases. It is characterized by an autosomal dominant mode of inheritance and by a highly increased risk of CRC and endometrial cancer, as well as to a lesser extent of some other cancers (e.g., ovary, stomach, small intestine, biliary and urinary tract) [4]. Germline pathogenic variants in the main DNA mismatch repair (MMR) genes, *MLH1*, *MSH2*, *MSH6* and *PMS2*, explain the majority of Lynch syndrome cases. Most Lynch syndrome-associated CRC tumors harbor a phenotype of high DNA microsatellite instability (MSI).

Up to 30% of CRC patients report having one or more relatives also diagnosed with the disease but are not consistent with one of the known inherited syndromes. Family history (FH) of CRC is a well-known risk factor for the development of the tumor [5-9], but its impact on clinico-pathological characteristics of CRC in patients is not entirely understood [10-13]. Also, it is not entirely clear how FH may affect CRC survival as the few studies performed on this topic have inconsistent findings [7,14-20].

CRC is a heterogeneous disease with a variable natural history according to TNM stage. There is growing evidence that CRC should be subdivided into different prognostic groups defined by robust molecular biomarker combinations and intratumor features of immune infiltration [21]. Such markers could guide the use of more or less aggressive treatment regimens and enable clinicians to balance expected outcomes against early and late therapeutic toxicities. This is particularly important in the case of locally advanced CRC (stages II and III), in which patients may be cured by surgery alone and only a portion derive benefit from adjuvant therapy. However, most molecular markers and immune-based studies have limited power, analyze small numbers of tumors (<500 in most studies), and only a few are population-based [22-24]. To date, although several molecular markers of outcome in CRC have been proposed, MSI status and mutations in oncogenes such as *KRAS* and *BRAF* are those most consistently used

For peer review only - http://bmjopen.bmj.com/site/about/guidelines.xhtml

in clinical practice. However, patient outcomes remain variable, and stratification according to prognosis needs to be improved.

We set up a population-based cohort of CRC patients and their first- and second degree-relatives (FDRs and SDRs, respectively) registered in the Canton of Geneva, to evaluate the role of FH and tumor biomarkers on patient outcome (loco-regional and distant recurrences, survival) considering relevant clinico-pathological features and treatments.

COHORTS DESCRIPTION

Cohort of CRC patients

The cohort of CRC patients has been established using the Geneva Cancer Registry (GCR) database which includes all incident cases of malignant neoplasms occurring since 1970 in the population of this Swiss canton (approximately 500,000 inhabitants).

Patients who were diagnosed with a primary invasive cancer of the colon, except appendix (International Classification of Diseases for Oncology ICD-O, C18.0, C18.2-C18.9), the rectosigmoid junction (ICD-O, C19) and the rectum (ICD-O, C20) between 1985 and 2013, were eligible for inclusion in the CRC cohort. For colon cancer, the splenic flexure was considered as the frontier to define right-sided and left-sided tumors. Thus, right colon cancers included tumors occurring in the cecum, ascending colon, hepatic flexure, and transverse colon (ICD-O codes: C18.0, C18.2-18.4). Left-sided tumors included tumors located at the splenic flexure, descending colon, sigmoid, and recto-sigmoid junction (ICD-O codes: C18.5-18.7, 19.9).

A prospective open-ended follow-up of all cancer cases registered in the GCR database since 1970 is realized annually to collect data on recurrences (loco-regional and distant), second cancer and survival. These ongoing follow-up data are registered in the Geneva Cancer Registry database. For the present report, we used data on events that occurred up to 31/Dec/2019 in the CRC cohort.

Overall, 5985 CRC cases were registered in the GCR database between 1985 and 2013. Of these, 486 (8.1%) cases were subsequently excluded (mainly cases discovered from the death certificate or at the autopsy (n=217), cases with *in situ* cancer, cancer of other sites, Bauhin valve cancer, lymphomas, sarcomas, melanomas, or no cancer (n=243), and lost medical charts (n=26). Accordingly, the final cohort population includes 5499 patients (4244 colon cancer (CC) patients and 1255 rectal cancer (RC) patients.

For peer review only - http://bmjopen.bmj.com/site/about/guidelines.xhtml

Cohort of relatives of CRC patients

Parents and offspring of CRC patients have been identified through a computerized search in the administrative files of the Cantonal Population Office (CPO). The CPO maintains a register in which names, date of birth and personal OCP identification number are recorded for all residents in the Canton of Geneva since 1985. Using the CPO identification number of the CRC patient included in the GCR dataset, we can identify the name, identification number, date of birth, and date of death or emigration (if applicable) of all first-degree "vertical" links, i.e. both the parents and the offspring. To trace siblings of the CRC patients, the search is done using as key the CPO identification number of the parents identified in the previous step. A similar linkage process allows identifying SDR (i.e., grandparents, aunts, uncles, grandchildren, nieces and nephews, half-brothers and half-sisters). An automated procedure to identify FDRs and SDRs is being developed. The cohort of relatives will be updated at the end of every year using the annually updated GCR and CPO files.

DATA COLLECTION

Cohort of CRC patients

Information collected for the 5499 CRC patients included in the cohort is summarized in Table 1.

Table 1. Summary of data collected for CRC patients and data source.

**Data collected routinely by the Geneva Cancer Registry**	
**Baseline**	
Demographics/Socioeconomics	Date and place of birth, gender, marital status, nationality, last occupation
Medical history of cancer	Dates, sites, tumor characteristics, treatments
Diagnostic circumstances	Method of detection, presence of symptoms, methods of assessment
Treatments (first 6 months after diagnosis)	Type of surgery, radiotherapy, chemotherapy, finality (curative or palliative), surgical margins
Sector of care	Public or private
Tumor characteristics	Histological type and subtype, differentiation, TNM stage
**Follow-up**	
Locoregional and distant recurrences	Dates, sites, tumor characteristics, treatments
Second cancer	Dates, sites, tumor characteristics, treatments
Death	Date, cause and place
**Data collected from medical files**	
**Baseline**	

For peer review only - http://bmjopen.bmj.com/site/about/guidelines.xhtml

Lifestyle	History of tobacco consumption, alcohol habits
Anthropometrics	Weight, height
Medical history	Comorbidities, previous gastrointestinal diseases
Medication use	Aspirin, metformin, statins, vitamin D
Family history	History of cancer among FDRs and SDRs
Procedures prior diagnostic	Fecal occult blood tests, endoscopies, imaging
Origin of diagnosis	Colonoscopy, recto-sigmoidoscopy, barium enema
Type of surgery	Elective or on emergency, presence of perforation
Tumor characteristics	Location, development of polyps, Dukes stage, lymphovascular invasion, necrosis
**Data collected from linkage with the administrative OCP database**	
FDRs and SDRs	Gender, dates of birth, dates of death
**Data collected from linkage with the Oncogenetic and Cancer Prevention Unit**	
Genetic counseling and germline testing	Uptake and results

Abbreviation: FDRs, first-degree relatives, SDRs, second-degree relatives.

For peer review only

Data on patient and tumor characteristics, treatment and outcomes were extracted from the GCR database. The registry systematically records sociodemographic information (date of birth, gender, nationality, place of birth, marital status, and occupation), diagnostic circumstances (origin of diagnosis, presence of symptoms, and methods of assessment), tumor characteristics (histologic type, differentiation coded according to ICD-O, stage of the disease at diagnosis (size of the tumor, cTNM, pTNM, SEER, number of lymph nodes resected, number of positive lymph nodes), treatment during the first 6 months after diagnosis (type of surgery, radiotherapy, chemotherapy, etc.), finality of the treatment (curative, palliative, or not specified), surgical margin of the tumor resection (positive, negative), occurrence of recurrences (loco-regional and distant), second tumor occurrence, and if applicable date and cause of death.

Additional clinical information including type and date of surgery (elective or on emergency), presence of perforation, presence of polyps and presence of comorbidities was collected in the clinical files of CRC patients. A structured questionnaire was developed for the study and data were entered online by 3 registrars into a central database specifically designed to store all data.

Data on genetic counseling uptake and germline testing was also be collected using a linkage procedure with the database established by the Oncogenetics Unit at University Hospitals of

For peer review only - http://bmjopen.bmj.com/site/about/guidelines.xhtml

Geneva, the only center providing genetic counseling for familial aggregation or CRC predisposition syndromes in the Geneva area since 1994.

Cohort of relatives of CRC patients

Information about cancer among relatives of CRC patients will be retrieved from the GCR dataset using their GCR identification number. All cancer diagnoses registered in the GCR since its creation in 1970 will be recorded.

Data on cancer occurrence in the families will be updated at the end of every year using the annually updated files of CPO and GCR.

Patient and public involvement

Patients and/or the public were not involved in the design, conduct, reporting or dissemination of this research.

SAMPLE COLLECTION AND NEXT-GENERATION TISSUE MICROARRAY (ngTMA) CONSTRUCTION

For each case, formalin-fixed paraffin-embedded (FFPE) tissue samples of the primary tumor and slides were searched in the pathology laboratories using biopsy/surgery report numbers extracted from pathology reports. FFPE blocks (excluding biopsies) were then collected and tissue reembedded in fresh paraffin blocks if necessary. From each block, a new section was cut, hematoxylin & eosin (HE) stained and scanned at high resolution on a whole slide scanner (Pannoramic 250 Flash II, 3DHistech). For ngTMA construction, four circular regions with 600µm diameter were defined on the whole slide image, two for tumor center and for two tumor border. Based on the coordinates defined by these regions, a ngTMA was constructed using a TMA Grand Master (3DHistech).

TMAs have been constructed for 3723 (67.7%) of the 5499 CRC patients. The tumors are being currently classified as microsatellite stable or instable on the basis of the expression of the main 4 DNA MMR proteins (MLH1, MSH2, MSH6, PMS2). Other biomarkers, such as the *BRAF* V600E mutation status and the Immunoscore [25] will be assessed. We expect to correlate data on microsatellite status with updated follow-up data in 2021 and those on BRAF mutation and Immunoscore with updated follow-up data in 2022.

For peer review only - http://bmjopen.bmj.com/site/about/guidelines.xhtml

FINDINGS TO DATE

Patient characteristics are shown in Table 2.

Table 2. Patient characteristics at first CRC diagnosis.

Patient characteristics	Colon cancer n=4244	Rectal cancer n=1255	Total n=5499
**Gender, n (%)**			
Male	2116 (49.9)	723 (57.6)	2839 (51.6)
Female	2128 (50.1)	532 (42.4)	2660 (48.4)
**Age at diagnosis (years), n (%)**			
< 50	234 (5.5)	96 (7.6)	330 (6.0)
50-59	568 (13.4)	221 (17.6)	789 (14.3)
60-69	948 (22.3)	346 (27.6)	1294 (23.5)
70-79	1275 (30.0)	336 (26.8)	1611 (29.3)
≥ 80	1219 (28.7)	256 (20.4)	1475 (26.8)
**Nationality, n (%)**			
Swiss	3241 (76.4)	909 (72.4)	4150 (75.5)
Other	1003 (23.6)	346 (27.6)	1349 (24.5)
**Marital status, n (%)**			
Married/Partnership	2289 (53.9)	724 (57.7)	3013 (54.8)
Single/Widowed/Divorced	1955 (46.1)	531 (42.3)	2486 (45.2)
**Socioeconomic status, n (%)**			
High	670 (15.8)	185 (14.7)	855 (15.5)
Medium	1679 (39.6)	515 (41.0)	2194 (39.9)
Low	1056 (24.9)	369 (29.4)	1425 (25.9)
Unclassifiable/Missing	839 (19.8)	186 (14.8)	1025 (18.6)
**Period of diagnosis, n (%)**			
1985-1989	666 (15.7)	180 (14.3)	846 (15.4)
1990-1994	678 (16.0)	216 (17.2)	894 (16.3)
1995-1999	722 (17.0)	221 (17.6)	943 (17.1)
2000-2004	735 (17.3)	207 (16.5)	942 (17.1)
2005-2009	803 (18.9)	235 (18.7)	1038 (18.9)
2010-2013	640 (15.1)	196 (15.6)	836 (15.2)
**Charlson comorbidity index, n (%)**			
0	1356 (32.0)	538 (42.9)	1894 (34.4)
1-2	982 (23.1)	279 (22.2)	1261 (22.9)
3-4	237 (5.6)	66 (5.3)	303 (5.5)
≥ 5	474 (11.2)	106 (8.4)	580 (10.5)
Missing	1195 (28.2)	266 (21.2)	1461 (26.6)
**Familial history of CRC, n (%)**			
No	1195 (28.2)	521 (41.5)	1716 (31.2)
Yes	249 (5.9)	115 (9.2)	364 (6.6)
Missing	2800 (66.0)	619 (49.3)	3419 (62.2)
**At least one genetic counseling uptake, n (%)**			
No	4160 (98.0)	1228 (97.8)	5388 (98.0)
Yes	84 (2.0)	27 (2.2)	111 (2.0)
**History of CRC in FDRs, n (%)**			
No	1195 (28.2)	521 (41.5)	1716 (31.2)
Yes	249 (5.9)	115 (9.2)	364 (6.6)
Missing	2800 (66.0)	619 (49.3)	3419 (62.2)
**Colon cancer location**			
Right colon	1731 (40.8)		
Left colon	1964 (46.3)		

For peer review only

Not specified/Overlap	549 (12.9)

Abbreviation: FDRs, first-degree relatives

The median age at diagnosis is 73 years (range: 16-101) for colon cancer (CC) patients and 68 years (range: 24-101) for rectal cancer (RC) patients. Approximately 50% of CC cases and 58% of RC cases are males. Among the 4244 patients with CC, 41% had right CC, 46% left CC, and 13% overlapping tumors or unknown CC location. Tumor and treatment characteristics of CRC patients are shown in Table 3.

Table 3. Tumor diagnosis and treatment characteristics at CRC diagnosis.

Tumor diagnosis/treatment	Colon cancer n=4244	Rectal cancer n=1255	Total n=5499
**Reason leading to diagnosis, n (%)**			
Population screening	136 (3.2)	37 (2.9)	173 (3.1)
High risk	39 (0.9)	16 (1.3)	55 (1.0)
Symptoms	3421 (80.6)	1083 (86.3)	4504 (81.9)
Fortuitous discovery	434 (10.2)	72 (5.7)	506 (9.2)
Others	18 (0.4)	6 (0.5)	24 (0.4)
Missing	196 (4.6)	41 (3.3)	237 (4.3)
**Abdominal emergency, n (%)**			
No	2466 (58.1)	976 (77.8)	3442 (62.6)
Yes	763 (18.0)	63 (5.0)	826 (15.0)
Missing	1015 (23.9)	216 (17.2)	1231 (22.4)
**Imaging use for diagnosis, n (%)**			
PET scan	16 (0.4)	4 (0.3)	20 (0.4)
CT scan/MRI	780 (18.4)	250 (19.9)	1030 (18.7)
Others	443 (10.4)	113 (9.0)	556 (10.1)
**Stage, n (%)**			
I	657 (15.5)	213 (17.0)	870 (15.8)
II	1262 (29.7)	275 (21.9)	1537 (28.0)
III	950 (22.4)	436 (34.7)	1385 (25.2)
IV	976 (23.0)	205 (16.3)	1182 (21.5)
Missing	399 (9.4)	126 (10.0)	525 (9.5)
**Differentiation, n (%)**			
Well differentiated	792 (18.7)	243 (19.4)	1035 (18.8)
Moderately differentiated	2264 (53.3)	681 (54.3)	2945 (53.6)
Poorly differentiated	563 (13.2)	111 (8.9)	674 (12.2)
Missing	625 (14.7)	220 (17.5)	845 (15.4)
**Blood vessels invasion, n (%)**			
No	2132 (50.2)	430 (34.3)	2562 (46.6)
Yes	886 (20.9)	186 (14.8)	1072 (19.5)
Missing	1226 (28.9)	639 (50.9)	1865 (33.9)
**Lymph node invasion, n (%)**			
No	2159 (50.9)	459 (36.6)	2618 (47.6)
Yes	612 (14.4)	89 (7.1)	701 (12.7)
Missing	1473 (34.7)	707 (56.3)	2180 (39.6)
**Surgery, n (%)**			
No	521 (12.3)	207 (16.5)	728 (13.2)
Yes	3698 (87.1)	1040 (82.9)	4738 (86.2)
Missing	25 (0.6)	8 (0.6)	33 (0.6)
**Chemotherapy, n (%)**			
No	3146 (74.1)	694 (55.3)	3840 (69.8)
Yes	934 (22.0)	518 (41.3)	1452 (26.4)
Missing	164 (3.9)	43 (3.4)	207 (3.8)
**Radiotherapy, n (%)**			

For peer review only

For peer review only - http://bmjopen.bmj.com/site/about/guidelines.xhtml

No	4131 (97.3)	629 (50.1)	4760 (88.6)
Yes	97 (2.3)	597 (47.6)	694 (12.6)
Missing	16 (0.4)	29 (2.3)	45 (0.8)

Only 3% of patients were diagnosed during a screening procedure. This low proportion is partly explained by the absence of a population screening program in Geneva and lack of reimbursement of screening exams before 2014. Thus, the great majority of both CC and RC patients were diagnosed because of occurrence of symptoms (80.6% and 86.3%, respectively). Abdominal emergency at diagnosis was 4-fold more frequent for CC patients than RC patients (18% versus 5%). Cancer diagnosis was histologically confirmed in 90% of cases. About half of CC and RC cases were diagnosed with an advanced disease, i.e. stages III and IV (Table 3). Tumors were surgically resected for 82% of CC cases and 87% of RC cases. RC patients were more often treated with chemotherapy and radiotherapy than CC patients.

At the end of 2019, 337 local recurrences, 1143 distant recurrences and 4035 deaths were reported. The median follow-up for the cohort was 4.5 years (Q1;1.1-Q3;10.5). Five percent of patients were lost during follow-up. CRC patients were followed for survival from the date of diagnosis to the date of death or emigration or end of 2019, whichever came first. We considered both the CRC-specific survival time, defined as the interval between the date of diagnosis and the date of death from CRC, and the overall survival time, defined as the interval between the date of diagnosis and the date of death from any cause. Specific-survival rates for colon and rectal cancers and overall survival rates were calculated using the Kaplan-Meier method. The 5-year disease-free survival rate was 48% (95% CI: 47%-50%) for CC and 51% (95% CI: 49%-54%) for RC patients. The 5-year overall survival rate was 49% (95% CI: 48%-51%) for CC and 53% (95% CI: 50%-56%) for RC patients. The 5-year cancer-specific survival rate was 61% (95%CI: 59%-62%) for CC and 63% (95%CI: 61%-66%) for RC patients (Figure 1). Specific-survival rates were significantly different (logrank test, $p<0.001$) between stages among patients with CC (Figure 2) and among patients with rectal cancer (Figure 3).

COLLABORATION

The Geneva Cancer Registry welcomes collaboration with national and international research groups. Study protocols can be proposed and submitted to the Geneva ethics committee if pertinent. In the case of collaborative studies, anonymized datasets in several formats can be shared by secure file transfer. For further information please contact the corresponding author.

FURTHER DETAILS

For peer review only - http://bmjopen.bmj.com/site/about/guidelines.xhtml

Contributors: SB, GP, FR, LRB, VV, TZ, IZ, POC, and ER contributed the original concept and design of the study. EF, GP, LRB, TMK, EF, and IZ supervised the data collection and sample management. EF developed the linkage procedures and conducted the statistical analyses. SB drafted the manuscript. All authors provided input and feedback on the paper drafts and approved the final version of the manuscript.

Funding: The study was supported by grants from the Swiss Science National Foundation (320030-163342/1), the Swiss Cancer League (KFS-3932-08-2016-R and KFS-5243-02-2021) and the Geneva Cancer League (subvention 1813).

Competing interests: The authors declare they have no conflict of interest.

Ethics approval: The study protocol has been approved by the Geneva Ethics Committee (Commission cantonale de la recherche scientifique de Genève [CCER], N° 2016-00141). During the study period, Geneva Cancer Registry hold all the necessary permissions to register the prognostic factors without written consent of cancer patients. Patients were informed that their data were recorded by the GCR and had the right to be ruled out from the database.

Patient consent for publication: Not required.

Data availability: Researchers may request access to data by contacting the corresponding author.

For peer review only

References

1. Ferlay J, Ervik M, Lam F, et al. Global Cancer Observatory: Cancer Today. Lyon, France: International Agency for Research on Cancer. Available from: https://gco.iarc.fr/today, accessed [13 April 2022]
2. Bishehsari F, Mahdavinia M, Vacca M, *et al*. Epidemiological transition of colorectal cancer in developing countries: environmental factors, molecular pathways, and opportunities for prevention. *World J Gastroenterol* 2014;20:6055-72.
3. Stoffel EM, Mangu PB, Gruber SB, et al. Hamilton SR, Kalady MF, Lau MW, et al. Hereditary colorectal cancer syndromes: American Society of clinical oncology clinical practice guideline endorsement of the familial risk-colorectal cancer: European society for medical oncology clinical practice guidelines. *J Clin Oncol* 2015;33:209-17.
4. Marra G, Boland CR. Hereditary nonpolyposis colorectal cancer: the syndrome, the genes, and historical perspectives. *J Natl Cancer Inst* 1995;87:1114-25.
5. Fuchs CS, Giovannucci EL, Colditz GA, *et al*. A prospective study of family history and the risk of colorectal cancer. *N Engl J Med* 1994;331:1669-74.
6. Johns LE, Houlston RS. A systematic review and meta-analysis of familial colorectal cancer risk. *Am J Gastroenterol* 2001;96:2992-3003.
7. Slattery ML, Kerber RA. Family history of cancer and colon cancer risk: the Utah Population Database. *J Natl Cancer Inst* 1994;86:1618-26.
8. Antelo M, Castells A. Family history of colorectal cancer: a new survival predictor of colon cancer? *Gastroenterology* 2009;136:357-9.
9. Butterworth AS, Higgins JP, Pharoah P. Relative and absolute risk of colorectal cancer for individuals with a family history: a meta-analysis. *Eur J Cancer* 2006;42:216-27.
10. Kerber RA, Slattery ML, Potter JD, *et al*. Risk of colon cancer associated with a family history of cancer or colorectal polyps: the diet, activity, and reproduction in colon cancer study. *Int J Cancer* 1998;78:157-60.
11. Aste H, Picasso M, Allegretti A, *et al*. Correlation between family history of colorectal cancer and pathological and clinical features of the disease. *Cancer Detect Prev* 1994;18:343-8.
12. Cannon-Albright LA, Thomas TC, Bishop DT, *et al*. Characteristics of familial colon cancer in a large population data base. *Cancer* 1989;64:1971-5.
13. No authors. Clinical and pathological analyses of patients with a family history of colorectal cancer. Registry Committee, Japanese Research Society for Cancer of the Colon and Rectum. *Jpn J Clin Oncol* 1993;23:342-9.
14. Slattery ML, Kerber RA. The impact of family history of colon cancer on survival after diagnosis with colon cancer. *Int J Epidemiol* 1995;24:888-96.
15. Chan JA, Meyerhardt JA, Niedzwiecki D, *et al*. Association of family history with cancer recurrence and survival among patients with stage III colon cancer. *JAMA* 2008;299:2515-23.
16. Kao PS, Lin JK, Wang HS, *et al*. The impact of family history on the outcome of patients with colorectal cancer in a veterans' hospital. *Int J Colorectal Dis* 2009;24:1249-54.
17. Zell JA, Honda J, Ziogas A, *et al*. Survival after colorectal cancer diagnosis is associated with colorectal cancer family history. *Cancer Epidemiol Biomarkers Prev* 2008;17:3134-40.
18. Bass AJ, Meyerhardt JA, Chan JA, *et al*. Family history and survival after colorectal cancer diagnosis. *Cancer* 2008;112:1222-9.
19. Kune GA, Kune S, Watson LF. The effect of family history of cancer, religion, parity and migrant status on survival in colorectal cancer. The Melbourne Colorectal Cancer Study. *Eur J Cancer* 1992;28A:1484-7.
20. Kirchhoff AC, Newcomb PA, Trentham-Dietz A, *et al*. Family history and colorectal cancer survival in women. *Fam Cancer* 2008;7:287-92.
21. Bruni D, Angell HK, Galon J. The immune contexture and Immunoscore in cancer prognosis and therapeutic efficacy. *Nature Rev Cancer* 2020;20:662-80.

For peer review only

For peer review only - http://bmjopen.bmj.com/site/about/guidelines.xhtml

22. Merok MA, Ahlquist T, Røyrvik EC, *et al*. Microsatellite instability has a positive prognostic impact on stage II colorectal cancer after complete resection: results from a large, consecutive Norwegian series. *Ann Oncol* 2013;24:1274-82.
23. Seppälä TT, Böhm JP, Friman M, *et al*. Combination of microsatellite instability and BRAF mutation status for subtyping colorectal cancer. *Br J Cancer* 2015;112:1966-75.
24. Samowitz WS, Curtin K, Ma KN, *et al*. Microsatellite instability in sporadic colon cancer is associated with an improved prognosis at the population level. *Cancer Epidemiol Biomarkers Prev* 2001;10:917-23.
25. Pagès F, Mlecnik B, Marliot F, Bindea G, Ou F-S, Bifulco C, et al. International validation of the consensus Immunoscore for the classification of colon cancer: a prognostic and accuracy study. *Lancet* 26 2018;391:2128-39.

For peer review only

For peer review only - http://bmjopen.bmj.com/site/about/guidelines.xhtml

Figure legends

Figure 1. Specific survival rates for colon cancer and rectal cancer

Figure 2. Specific survival rates for colon cancer according to stages

Figure 3. Specific survival rates for rectal cancer according to stages

For peer review only

For peer review only - http://bmjopen.bmj.com/site/about/guidelines.xhtml

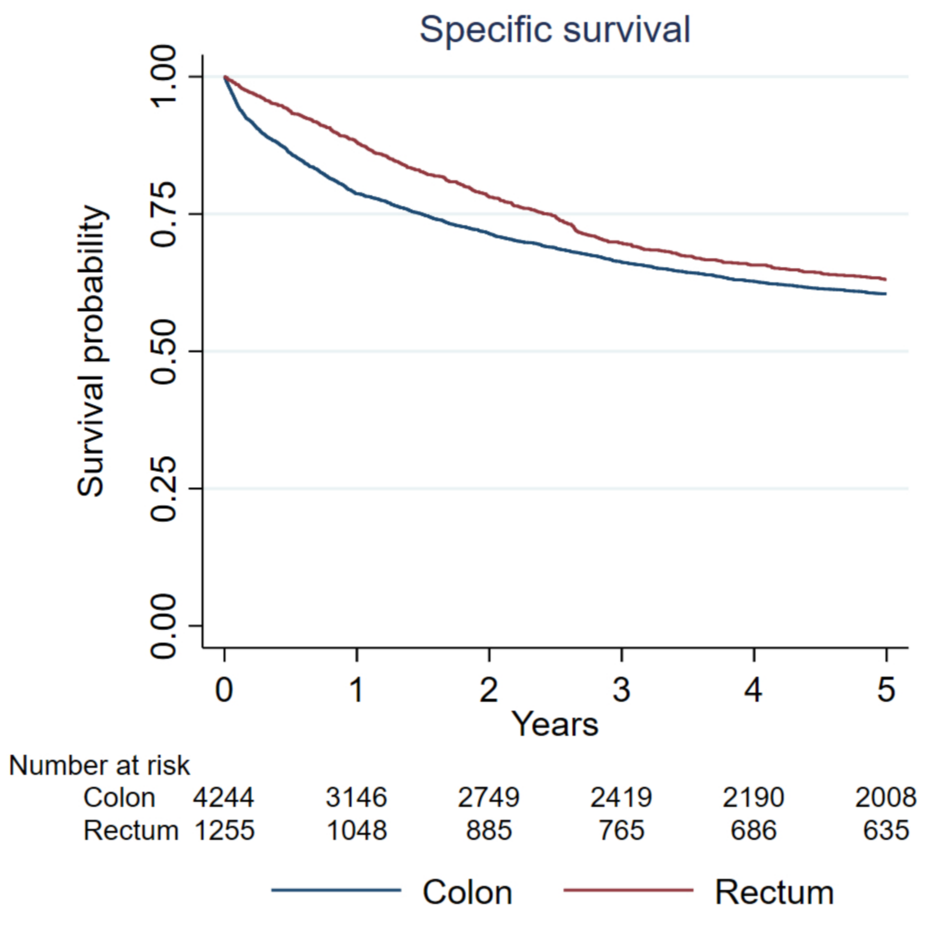

84x84mm (300 x 300 DPI)

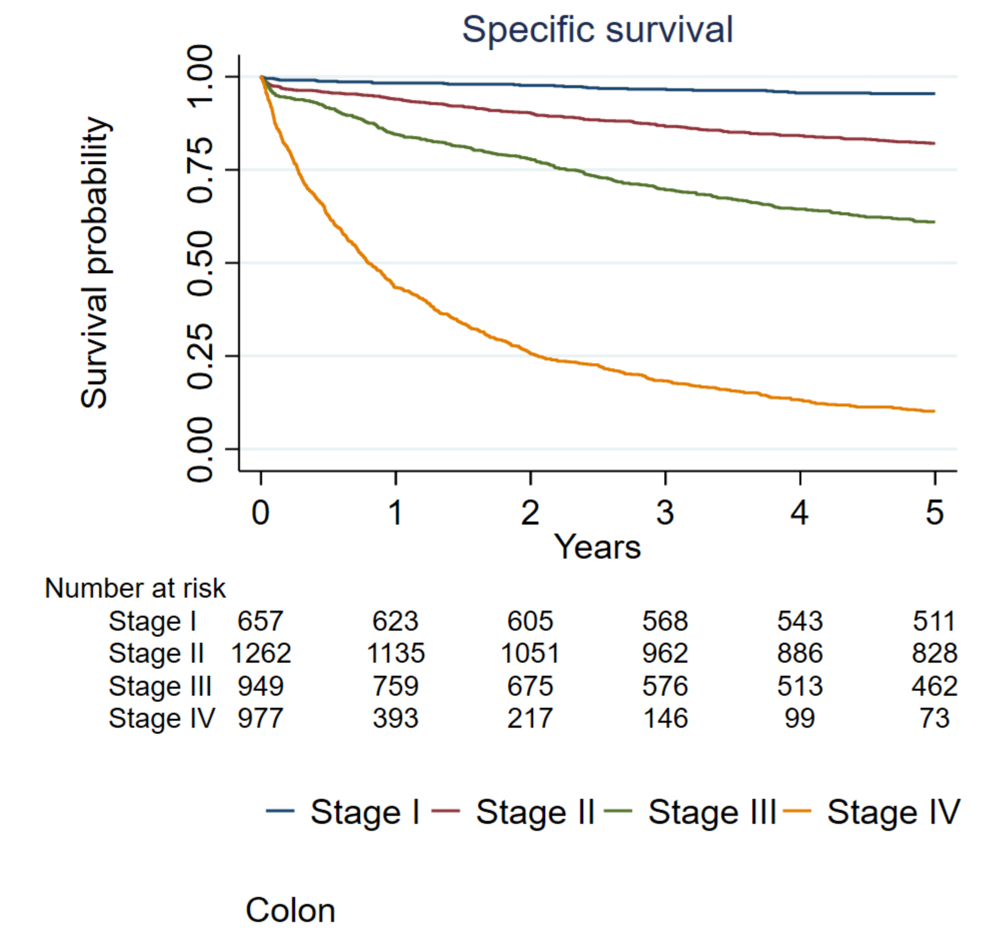

Number at risk

	0	1	2	3	4	5
Stage I	657	623	605	568	543	511
Stage II	1262	1135	1051	962	886	828
Stage III	949	759	675	576	513	462
Stage IV	977	393	217	146	99	73

84x84mm (300 x 300 DPI)

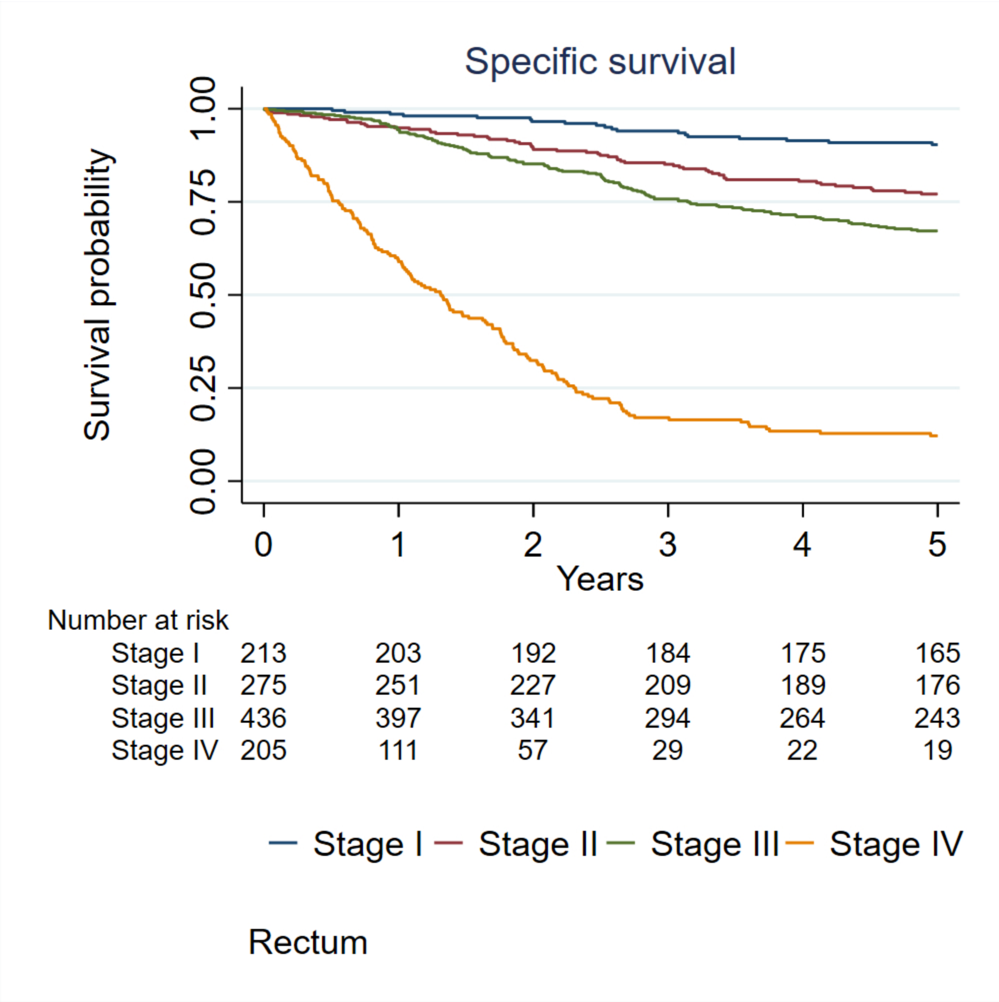

84x84mm (300 x 300 DPI)

BMJ Open

Cohort profile: population-based cohorts of patients with colorectal cancer and of their relatives in Geneva, Switzerland

Journal:	*BMJ Open*
Manuscript ID	bmjopen-2022-063914.R1
Article Type:	Cohort profile
Date Submitted by the Author:	15-Jul-2022
Complete List of Authors:	Benhamou, Simone; University of Geneva, Global Health Institute, Geneva Cancer Registry; Research Centre on Epidemiology and Population Health, INSERM Unit 1018<br>Fournier, Evelyne; University of Geneva, Global Health Institute, Geneva Cancer Registry<br>Puppa, Giacomo; Geneva University Hospitals, Department of Clinical Pathology<br>McKee, Thomas; Geneva University Hospitals, Department of Clinical Pathology<br>Ris, Frédéric; Geneva University Hospitals, Division of Digestive Surgery<br>Rubbia-Brandt, Laura; Geneva University Hospitals, Department of Clinical Pathology<br>Viassolo, Valeria; Geneva University Hospitals, Oncogenetics Unit, Division of Oncology; Geneva University Hospitals, Division of Genetic Medicine<br>Zilli, Thomas; Geneva University Hospitals, Radiation Oncology; University of Geneva, Faculty of Medicine<br>Zlobec, Inti; University of Bern, Institute of Pathology<br>Chappuis, Pierre; Geneva University Hospitals, Oncogenetics Unit, Division of Oncology; Geneva University Hospitals, Division of Genetic Medicine<br>Rapiti, Elisabetta; University of Geneva, Geneva Cancer Registry, Global Health Institute
**Primary Subject Heading**:	Epidemiology
Secondary Subject Heading:	Gastroenterology and hepatology, Public health, Oncology
Keywords:	EPIDEMIOLOGY, Gastrointestinal tumours < GASTROENTEROLOGY, PUBLIC HEALTH

SCHOLARONE™ Manuscripts

I, the Submitting Author has the right to grant and does grant on behalf of all authors of the Work (as defined in the below author licence), an exclusive licence and/or a non-exclusive licence for contributions from authors who are: i) UK Crown employees; ii) where BMJ has agreed a CC-BY licence shall apply, and/or iii) in accordance with the terms applicable for US Federal Government officers or employees acting as part of their official duties; on a worldwide, perpetual, irrevocable, royalty-free basis to BMJ Publishing Group Ltd ("BMJ") its licensees and where the relevant Journal is co-owned by BMJ to the co-owners of the Journal, to publish the Work in this journal and any other BMJ products and to exploit all rights, as set out in our licence.

The Submitting Author accepts and understands that any supply made under these terms is made by BMJ to the Submitting Author unless you are acting as an employee on behalf of your employer or a postgraduate student of an affiliated institution which is paying any applicable article publishing charge ("APC") for Open Access articles. Where the Submitting Author wishes to make the Work available on an Open Access basis (and intends to pay the relevant APC), the terms of reuse of such Open Access shall be governed by a Creative Commons licence – details of these licences and which Creative Commons licence will apply to this Work are set out in our licence referred to above.

Other than as permitted in any relevant BMJ Author's Self Archiving Policies, I confirm this Work has not been accepted for publication elsewhere, is not being considered for publication elsewhere and does not duplicate material already published. I confirm all authors consent to publication of this Work and authorise the granting of this licence.

For peer review only

Cohort profile: population-based cohorts of patients with colorectal cancer and of their relatives in Geneva, Switzerland

Simone Benhamou [1,2*], Evelyne Fournier[1], Giacomo Puppa[3], Thomas McKee[3], Frédéric Ris[4], Laura Rubbia-Brandt[3], Valeria Viassolo[5,6], Thomas Zilli[7], Inti Zlobec[8], Pierre Olivier Chappuis[5,6], Elisabetta Rapiti[1]

[1] Geneva Cancer Registry, Global Health Institute, University of Geneva, Geneva, Switzerland

[2] Research Centre in Epidemiology and Population Health, INSERM Unit 1018, Villejuif, France

[3] Department of Clinical Pathology, Geneva University Hospitals, Geneva, Switzerland

[4] Division of Digestive Surgery, Geneva University Hospitals, Genève 1211, Switzerland

[5] Oncogenetics Unit, Division of Oncology, Geneva University Hospitals, Geneva, Switzerland

[6] Division of Genetic Medicine, Geneva University Hospitals, Geneva, Switzerland

[7] Radiation Oncology, Geneva University Hospital, Geneva, Switzerland, and Faculty of Medicine, Geneva University, Geneva, Switzerland

[8] Institute of Pathology, University of Bern, Murtenstrasse 31, 3008, Switzerland

*Correspondence to:
Professor Simone Benhamou
Geneva Cancer Registry,
Global Health Institute,
University of Geneva, Geneva, Switzerland
simone.benhamou@inserm.fr

For peer review only - http://bmjopen.bmj.com/site/about/guidelines.xhtml

ABSTRACT

Purpose: Colorectal cancer is the 3rd leading cause of cancer death worldwide. Variability between patients in prognosis and treatment response is partially explained by traditional clinico-pathological factors. We established a large population-based cohort of patients with colorectal cancer and their first- and second-degree relatives registered in the Canton of Geneva, to evaluate the role of family history and tumor biomarkers on patient outcomes.

Participants: The cohort includes all patients with colorectal cancer diagnosed between 1985 and 2013. Detailed information on patient and tumor characteristics, treatment and outcomes were extracted from the Geneva Cancer Registry database, completed by medical records review, and linkage with administrative and oncogenetics databases. Next-generation tissue microarrays were constructed from tissue samples of the primary tumor. A prospective follow-up of the cohort is realized annually to collect data on outcomes. First- and second-degree relatives of patients are identified through linkage with the Cantonal Population Office database and information about cancer among relatives is retrieved from the Geneva Cancer Registry database. The cohort of relatives is updated annually.

Findings to date: The cohort includes 5499 patients (4244 patients with colon cancer and 1255 patients with rectal cancer). The great majority of patients were diagnosed because of occurrence of symptoms and almost half of cases were diagnosed with an advanced disease. At the end of 2019, 337 local recurrences, 1143 distant recurrences, and 4035 deaths were reported. At the same date, the cohort of first-degree relatives included 344 fathers, 538 mothers, 3485 children, and 375 siblings. Among them, we identified 28 fathers, 31 mothers, 18 siblings and 53 children who had a diagnosis of colorectal cancer.

Future plans: The cohort will be used for long-term studies of colorectal cancer epidemiology with focus on clinico-pathological factors and molecular markers. These data will be correlated with the most up-to-date follow-up data.

For peer review only - http://bmjopen.bmj.com/site/about/guidelines.xhtml

STRENGTHS AND LIMITATIONS OF THIS STUDY

- This study, using a powerful resource based on regional record-linkage analysis of routinely collected data, will allow having one of the most comprehensive cohorts of patients with CRC, and their relatives, at a population level.

- Considerable insights into the natural history of CRC and the mechanisms through which epidemiological, clinicopathological, and molecular markers may alter the prognosis are expected.
- Results of the study will provide evidence to guide health professionals in the stratification and management of patients with this disease, and surveillance of their relatives.
- A weakness of the cohort is the absence of tumor samples for approximately 30% of patients with CRC.
- Another limitation is the large amount of missing data for some of the recorded variables that will be addressed using multiple imputation procedures.

INTRODUCTION

In Europe, colorectal cancer (CRC) is the second most common malignancy in women and the third one in men [1]. In 2020, 281,714 men and 238,106 women were diagnosed with CRC, and it accounted for approximately 13% of all cancer-related deaths in both genders. More than 80% of all CRC occur at the age of 60 years or more.

In high-income countries, the risk of CRC has been ubiquitously associated with environmental factors, such as dietary patterns (diet rich in fat and meat and poor in unrefined cereals and fiber), obesity, and sedentary lifestyle [2].

Approximately 6% of patients with CRC carry germline mutations in genes responsible of inherited genetic predisposition to CRC [3]). Lynch syndrome is the most common hereditary CRC syndrome and has been implicated in 2% to 5% of newly diagnosed CRC cases. It is characterized by an autosomal dominant mode of inheritance and by a highly increased risk of CRC and endometrial cancer, as well as to a lesser extent of some other cancers (e.g., ovary, stomach, small intestine, biliary and urinary tract) [4]. Germline pathogenic variants in the main DNA mismatch repair (MMR) genes, *MLH1*, *MSH2*, *MSH6* and *PMS2*, explain the majority of Lynch syndrome cases. Most Lynch syndrome-associated CRC tumors harbor a phenotype of high DNA microsatellite instability (MSI).

Up to 30% of patients with CRC report having one or more relatives also diagnosed with the disease but are not consistent with one of the known inherited syndromes. Family history (FH)

For peer review only - http://bmjopen.bmj.com/site/about/guidelines.xhtml

of CRC is a well-known risk factor for the development of the tumor [5-9], but its impact on clinico-pathological characteristics of CRC in patients is not entirely understood [10-13]. Also, it is not entirely clear how FH may affect CRC survival as the few studies performed on this topic have inconsistent findings [7,14-20].

CRC is a heterogeneous disease with a variable natural history according to TNM stage. There is growing evidence that CRC should be subdivided into different prognostic groups defined by robust molecular biomarker combinations and intratumor features of immune infiltration [21]. Such markers could guide the use of more or less aggressive treatment regimens and enable clinicians to balance expected outcomes against early and late therapeutic toxicities. This is particularly important in the case of locally advanced CRC (stages II and III), in which patients may be cured by surgery alone and only a portion derive benefit from adjuvant therapy. However, most molecular markers and immune-based studies have limited power, analyze small numbers of tumors (<500 in most studies), and only a few are population-based [22-24]. To date, although several molecular markers of outcome in CRC have been proposed, MSI status and mutations in oncogenes such as *KRAS* and *BRAF* are those most consistently used in clinical practice. However, patient outcomes remain variable, and stratification according to prognosis needs to be improved.

We set up a population-based cohort of patients with CRC and their first- and second degree-relatives (FDRs and SDRs, respectively) registered in the Canton of Geneva, to evaluate the role of FH and tumor biomarkers on patient outcome (loco-regional and distant recurrences, survival) considering relevant clinico-pathological features and treatments.

COHORTS DESCRIPTION

Cohort of patients with CRC

The cohort of patients with CRC has been established using the Geneva Cancer Registry (GCR) database which includes all incident cases of malignant neoplasms occurring since 1970 in the population of this Swiss canton (approximately 500,000 inhabitants).

Patients who were diagnosed with a primary invasive cancer of the colon, except appendix (International Classification of Diseases for Oncology ICD-O, C18.0, C18.2-C18.9), the rectosigmoid junction (ICD-O, C19) and the rectum (ICD-O, C20) between 1985 and 2013, were eligible for inclusion in the CRC cohort. For colon cancer, the splenic flexure was considered as the frontier to define right-sided and left-sided tumors. Thus, right colon cancers included tumors occurring in the cecum, ascending colon, hepatic flexure, and transverse colon (ICD-O codes: C18.0, C18.2-18.4). Left-sided tumors included tumors located at the

For peer review only - http://bmjopen.bmj.com/site/about/guidelines.xhtml

splenic flexure, descending colon, sigmoid, and recto-sigmoid junction (ICD-O codes: C18.5-18.7, 19.9).

A prospective open-ended follow-up of all cancer cases registered in the GCR database since 1970 is realized annually to collect data on recurrences (loco-regional and distant), second cancer and survival. These ongoing follow-up data are registered in the Geneva Cancer Registry database. For the present report, we used data on events that occurred up to 31/Dec/2019 in the CRC cohort.

Overall, 5985 CRC cases were registered in the GCR database between 1985 and 2013. Of these, 486 (8.1%) cases were subsequently excluded (mainly cases discovered from the death certificate or at the autopsy (n=217), cases with *in situ* cancer, cancer of other sites, Bauhin valve cancer, lymphomas, sarcomas, melanomas, or no cancer (n=243), and lost medical charts (n=26). Accordingly, the final cohort population includes 5499 patients (4244 patients with colon cancer (CC) and 1255 patients with rectal cancer (RC).

Cohort of relatives of patients with CRC

Parents and offspring of patients with CRC have been identified through a computerized search in the administrative files of the Cantonal Population Office (CPO). The CPO maintains a register in which names, date of birth and personal OCP identification number are recorded for all residents in the Canton of Geneva since 1985. Using the CPO identification number of the CRC patient included in the GCR dataset, we can identify the name, identification number, date of birth, and date of death or emigration (if applicable) of all first-degree “vertical” links, i.e. both the parents and the offspring. To trace siblings of the patients with CRC, the search is done using as key the CPO identification number of the parents identified in the previous step. A similar linkage process allows identifying SDR (i.e., grandparents, aunts, uncles, grandchildren, nieces and nephews, half-brothers and half-sisters). An automated procedure to identify SDRs is being developed. The cohort of relatives will be updated at the end of every year using the annually updated GCR and CPO files.

Sources of data and links between the datasets are shown in Figure 1.

DATA COLLECTION

Cohort of patients with CRC

Information collected for the 5499 patients with CRC included in the cohort is summarized in Table 1.

For peer review only - http://bmjopen.bmj.com/site/about/guidelines.xhtml

Table 1. Summary of data collected for patients with CRC and data source

**Data collected routinely by the Geneva Cancer Registry**	
**Baseline**	
Demographics/Socioeconomics	Date and place of birth, gender, marital status, nationality, last occupation
Medical history of cancer	Dates, sites, tumor characteristics, treatments
Diagnostic circumstances	Method of detection, presence of symptoms, methods of assessment
Treatments (first 6 months after diagnosis)	Type of surgery, radiotherapy, chemotherapy, finality (curative or palliative), surgical margins
Sector of care	Public or private
Tumor characteristics	Histological type and subtype, differentiation, TNM stage
**Follow-up**	
Locoregional and distant recurrences	Dates, sites, tumor characteristics, treatments
Second cancer	Dates, sites, tumor characteristics, treatments
Death	Date, cause and place
**Data collected from medical files**	
**Baseline**	
Lifestyle	History of tobacco consumption, alcohol habits
Anthropometrics	Weight, height
Medical history	Comorbidities, previous gastrointestinal diseases
Medication use	Aspirin, metformin, statins, vitamin D
Family history	History of cancer among FDRs and SDRs
Procedures prior diagnostic	Fecal occult blood tests, endoscopies, imaging
Origin of diagnosis	Colonoscopy, recto-sigmoidoscopy, barium enema
Type of surgery	Elective or on emergency, presence of perforation
Tumor characteristics	Location, development of polyps, Dukes stage, lymphovascular invasion, necrosis
**Data collected from linkage with the administrative OCP database**	
FDRs and SDRs	Gender, dates of birth, dates of death
**Data collected from linkage with the Oncogenetics Unit database**	
Genetic counseling and germline testing	Uptake and results

Abbreviation: FDRs, first-degree relatives, SDRs, second-degree relatives.

Data on patient and tumor characteristics, treatment and outcomes were extracted from the GCR database. The registry systematically records sociodemographic information (date of

For peer review only

For peer review only - http://bmjopen.bmj.com/site/about/guidelines.xhtml

birth, gender, nationality, place of birth, marital status, and occupation), diagnostic circumstances (origin of diagnosis, presence of symptoms, and methods of assessment), tumor characteristics (histologic type, differentiation coded according to ICD-O, stage of the disease at diagnosis (size of the tumor, cTNM, pTNM, Surveillance, Epidemiology, and End Results, number of lymph nodes resected, number of positive lymph nodes), treatment during the first 6 months after diagnosis (type of surgery, radiotherapy, chemotherapy, etc.), finality of the treatment (curative, palliative, or not specified), surgical margin of the tumor resection (positive, negative), occurrence of recurrences (loco-regional and distant), second tumor occurrence, and if applicable date and cause of death.

Additional clinical information including type and date of surgery (elective or on emergency), presence of perforation, presence of polyps and presence of comorbidities was collected in the clinical files of patients with CRC. A structured questionnaire was developed for the study and data were entered online by 3 registrars into a central database specifically designed to store all data.

Data on genetic counseling uptake and germline testing was also collected using a linkage procedure with the database established by the Oncogenetics Unit at University Hospitals of Geneva, the only center providing genetic counseling for familial aggregation or CRC predisposition syndromes in the Geneva area since 1994.

Cohort of relatives of patients with CRC

Information about cancer among relatives of patients with CRC will be retrieved from the GCR dataset using their GCR identification number. All cancer diagnoses registered in the GCR since its creation in 1970 will be recorded.

Data on cancer occurrence in the families will be updated at the end of every year using the annually updated files of CPO and GCR.

Patient and public involvement

Patients and/or the public were not involved in the design and conduct of this research. The Steering Committee of the cohort is currently in the process of defining the procedures to access the resources of the cohorts (data and/or biological samples) by public entities for conducting specific research projects. One patient with colorectal cancer will be included in an extended Committee. All regulatory procedures will be outlined in a charter that we will make available to project leaders.

For peer review only

SAMPLE COLLECTION AND NEXT-GENERATION TISSUE MICROARRAY (ngTMA) CONSTRUCTION

For each case, formalin-fixed paraffin-embedded (FFPE) tissue samples of the primary tumor and slides were searched in the pathology laboratories using biopsy/surgery report numbers extracted from pathology reports. FFPE blocks (excluding biopsies) were then collected and tissue reembedded in fresh paraffin blocks if necessary. From each block, a new section was cut, hematoxylin & eosin (HE) stained and scanned at high resolution on a whole slide scanner (Pannoramic 250 Flash II, 3DHistech). For ngTMA construction, four circular regions with 600µm diameter were defined on the whole slide image, two for tumor center and for two tumor border. Based on the coordinates defined by these regions, a ngTMA was constructed using a TMA Grand Master (3DHistech).

TMAs have been constructed for 3723 (67.7%) of the 5499 patients with CRC. The tumors are being currently classified as microsatellite stable or instable on the basis of the expression of the main 4 DNA MMR proteins (MLH1, MSH2, MSH6, PMS2). Since 2014 these markers are analyzed and reported routinely for each patient diagnosed with CRC. Immunohistochemical loss of expression of MLH1, MSH2, MSH6 and/or PMS2 expression will be used to suspect Lynch syndrome. Additional analysis and information (*BRAF* mutation status, methylation of *MLH1* promoter, family history, germline testing) will be necessary to confirm or exclude this predisposition syndrome to hereditary cancer. Other biomarkers, such as the *BRAF* V600E mutation status and the Immunoscore [25] will be assessed. We expect to correlate data on microsatellite status with updated follow-up data in 2021 and those on BRAF mutation and Immunoscore with updated follow-up data in 2022.

FINDINGS TO DATE

Patient characteristics are shown in Table 2.

Table 2. Patient characteristics at first CRC diagnosis

Patient characteristics	Colon cancer n=4244	Rectal cancer n=1255	Total n=5499
**Gender, n (%)**			
Male	2116 (49.9)	723 (57.6)	2839 (51.6)
Female	2128 (50.1)	532 (42.4)	2660 (48.4)
**Age at diagnosis (years), n (%)**			
< 50	234 (5.5)	96 (7.6)	330 (6.0)
50-59	568 (13.4)	221 (17.6)	789 (14.3)
60-69	948 (22.3)	346 (27.6)	1294 (23.5)

For peer review only - http://bmjopen.bmj.com/site/about/guidelines.xhtml

70-79	1275 (30.0)	336 (26.8)	1611 (29.3)
≥ 80	1219 (28.7)	256 (20.4)	1475 (26.8)
**Nationality, n (%)**			
Swiss	3241 (76.4)	909 (72.4)	4150 (75.5)
Other	1003 (23.6)	346 (27.6)	1349 (24.5)
**Marital status, n (%)**			
Married/Partnership	2289 (53.9)	724 (57.7)	3013 (54.8)
Single/Widowed/Divorced	1955 (46.1)	531 (42.3)	2486 (45.2)
**Socioeconomic status, n (%)**			
High	670 (15.8)	185 (14.7)	855 (15.5)
Medium	1679 (39.6)	515 (41.0)	2194 (39.9)
Low	1056 (24.9)	369 (29.4)	1425 (25.9)
Unclassifiable/Missing	839 (19.8)	186 (14.8)	1025 (18.6)
**Period of diagnosis, n (%)**			
1985-1989	666 (15.7)	180 (14.3)	846 (15.4)
1990-1994	678 (16.0)	216 (17.2)	894 (16.3)
1995-1999	722 (17.0)	221 (17.6)	943 (17.1)
2000-2004	735 (17.3)	207 (16.5)	942 (17.1)
2005-2009	803 (18.9)	235 (18.7)	1038 (18.9)
2010-2013	640 (15.1)	196 (15.6)	836 (15.2)
**Charlson comorbidity index, n (%)**			
0	1356 (32.0)	538 (42.9)	1894 (34.4)
1-2	982 (23.1)	279 (22.2)	1261 (22.9)
3-4	237 (5.6)	66 (5.3)	303 (5.5)
≥ 5	474 (11.2)	106 (8.4)	580 (10.5)
Missing	1195 (28.2)	266 (21.2)	1461 (26.6)
**Familial history of CRC, n (%)**			
No	1195 (28.2)	521 (41.5)	1716 (31.2)
Yes	249 (5.9)	115 (9.2)	364 (6.6)
Missing	2800 (66.0)	619 (49.3)	3419 (62.2)
**At least one genetic counseling uptake, n (%)**			
No	4160 (98.0)	1228 (97.8)	5388 (98.0)
Yes	84 (2.0)	27 (2.2)	111 (2.0)
**History of CRC in FDRs, n (%)**			
No	1195 (28.2)	521 (41.5)	1716 (31.2)
Yes	249 (5.9)	115 (9.2)	364 (6.6)
Missing	2800 (66.0)	619 (49.3)	3419 (62.2)
**Colon cancer location**			
Right colon	1731 (40.8)		
Left colon	1964 (46.3)		
Not specified/Overlap	549 (12.9)		

Abbreviation: FDRs, first-degree relatives

The median age at diagnosis is 73 years (range: 16-101) for patients with colon cancer (CC) and 68 years (range: 24-101) for patients with rectal cancer (RC). Approximately 50% of cases with CC and 58% of cases with RC are males. Among the 4244 patients with CC, 41% had right CC, 46% left CC, and 13% overlapping tumors or unknown CC location. Tumor and treatment characteristics of patients with CRC are shown in Table 3.

Table 3. Tumor diagnosis and treatment characteristics at CRC diagnosis

Tumor diagnosis/treatment	Colon cancer n=4244	Rectal cancer n=1255	Total n=5499
**Reason leading to diagnosis, n (%)**			
Population screening	136 (3.2)	37 (2.9)	173 (3.1)
High risk	39 (0.9)	16 (1.3)	55 (1.0)

For peer review only

For peer review only - http://bmjopen.bmj.com/site/about/guidelines.xhtml

Symptoms	3421 (80.6)	1083 (86.3)	4504 (81.9)
Fortuitous discovery	434 (10.2)	72 (5.7)	506 (9.2)
Others	18 (0.4)	6 (0.5)	24 (0.4)
Missing	196 (4.6)	41 (3.3)	237 (4.3)
**Abdominal emergency, n (%)**			
No	2466 (58.1)	976 (77.8)	3442 (62.6)
Yes	763 (18.0)	63 (5.0)	826 (15.0)
Missing	1015 (23.9)	216 (17.2)	1231 (22.4)
**Imaging use for diagnosis, n (%)**			
PET scan	16 (0.4)	4 (0.3)	20 (0.4)
CT scan/MRI	780 (18.4)	250 (19.9)	1030 (18.7)
Others	443 (10.4)	113 (9.0)	556 (10.1)
**Stage, n (%)**			
I	657 (15.5)	213 (17.0)	870 (15.8)
II	1262 (29.7)	275 (21.9)	1537 (28.0)
III	950 (22.4)	436 (34.7)	1385 (25.2)
IV	976 (23.0)	205 (16.3)	1182 (21.5)
Missing	399 (9.4)	126 (10.0)	525 (9.5)
**Differentiation, n (%)**			
Well differentiated	792 (18.7)	243 (19.4)	1035 (18.8)
Moderately differentiated	2264 (53.3)	681 (54.3)	2945 (53.6)
Poorly differentiated	563 (13.2)	111 (8.9)	674 (12.2)
Missing	625 (14.7)	220 (17.5)	845 (15.4)
**Blood vessels invasion, n (%)**			
No	2132 (50.2)	430 (34.3)	2562 (46.6)
Yes	886 (20.9)	186 (14.8)	1072 (19.5)
Missing	1226 (28.9)	639 (50.9)	1865 (33.9)
**Lymph node invasion, n (%)**			
No	2159 (50.9)	459 (36.6)	2618 (47.6)
Yes	612 (14.4)	89 (7.1)	701 (12.7)
Missing	1473 (34.7)	707 (56.3)	2180 (39.6)
**Surgery, n (%)**			
No	521 (12.3)	207 (16.5)	728 (13.2)
Yes	3698 (87.1)	1040 (82.9)	4738 (86.2)
Missing	25 (0.6)	8 (0.6)	33 (0.6)
**Chemotherapy, n (%)**			
No	3146 (74.1)	694 (55.3)	3840 (69.8)
Yes	934 (22.0)	518 (41.3)	1452 (26.4)
Missing	164 (3.9)	43 (3.4)	207 (3.8)
**Radiotherapy, n (%)**			
No	4131 (97.3)	629 (50.1)	4760 (88.6)
Yes	97 (2.3)	597 (47.6)	694 (12.6)
Missing	16 (0.4)	29 (2.3)	45 (0.8)

Only 3% of patients were diagnosed during a screening procedure. This low proportion is partly explained by the absence of a population screening program in Geneva and lack of reimbursement of screening exams before 2014. Thus, the great majority of patients with CC or RC were diagnosed because of occurrence of symptoms (80.6% and 86.3%, respectively). Abdominal emergency at diagnosis was 4-fold more frequent for patients with CC than patients with RC (18% versus 5%). Cancer diagnosis was histologically confirmed in 90% of cases. About half of patients with CC or RC were diagnosed with an advanced disease, i.e. stages III and IV (Table 3). Tumors were surgically resected for 82% of cases with CC and 87% of cases with RC. Patients with RC were more often treated with chemotherapy and radiotherapy than patients with CC.

For peer review only - http://bmjopen.bmj.com/site/about/guidelines.xhtml

For peer review only

At the end of 2019, 337 local recurrences, 1143 distant recurrences and 4035 deaths were reported. The median follow-up for the cohort was 4.5 years (Q1;1.1-Q3;10.5). Five percent of patients were lost during follow-up. Patients with CRC were followed for survival from the date of diagnosis to the date of death or emigration or end of 2019, whichever came first. We considered both the CRC-specific survival time, defined as the interval between the date of diagnosis and the date of death from CRC, and the overall survival time, defined as the interval between the date of diagnosis and the date of death from any cause. Specific-survival rates for colon and rectal cancers and overall survival rates were calculated using the Kaplan-Meier method. The 5-year disease-free survival rate was 48% (95% CI: 47%-50%) for CC and 51% (95% CI: 49%-54%) for RC patients. The 5-year overall survival rate was 49% (95% CI: 48%-51%) for patients with CC and 53% (95% CI: 50%-56%) for patients with RC. The 5-year cancer-specific survival rate was 61% (95%CI: 59%-62%) for patients with CC and 63% (95%CI: 61%-66%) for patients with RC (Figure 2). Specific-survival rates were significantly different (logrank test, $p<0.001$) between stages among patients with CC (Figure 3) and among patients with RC (Figure 4).

At the end of 2019, the cohort of first-degree relatives included 344 fathers, 538 mothers, 3485 children, and 375 siblings of the 5499 patients with colorectal cancer. Among these family members, we identified 28 fathers, 31 mothers, 18 siblings and 53 children who had a diagnosis of colorectal cancer.

STRENGTHS AND LIMITATIONS

This study, using a powerful resource based on regional record-linkage analysis of routinely collected data will allow having, for each of CRC patient, the completeness of his/her FDR and SDR (identified by linkage with administrative population database) and the opportunity to identify in this cohort of relatives all those diagnosed with cancer by linkage with cancer registry data). Considerable insights into the natural history of CRC and the mechanisms through which epidemiological, clinic-pathological, and molecular markers may alter the prognosis are expected. Results of the study will provide evidence to guide health professionals in the stratification and management of patients with this disease, and surveillance of their relatives. Weaknesses of the cohort are the large amount of missing data for some of the recorded variables and the absence of tumor samples for approximately 30% of patients with CRC. We will use multiple imputation procedures to address the issue of missing data.

For peer review only - http://bmjopen.bmj.com/site/about/guidelines.xhtml

COLLABORATION

The Geneva Cancer Registry welcomes collaboration with national and international research groups to evaluate the role of FH and tumor biomarkers on patient outcome (loco-regional and distant recurrences, survival) considering relevant clinico-pathological features and treatments.

The Steering Committee of the cohort is currently in the process of defining the procedures to access the resources of the cohorts (data and/or biological samples) by public entities for conducting specific research projects. One patient with colorectal cancer will be included in an extended Committee. All regulatory procedures will be outlined in a charter that we will make available upon request to project leaders.

Contributors: SB, GP, FR, LRB, VV, TZ, IZ, POC, and ER contributed the original concept and design of the study. EF, GP, LRB, TMK, EF, and IZ supervised the data collection and sample management. EF developed the linkage procedures and conducted the statistical analyses. SB drafted the manuscript. All authors provided input and feedback on the paper drafts and approved the final version of the manuscript.

Funding: The study was supported by grants from the Swiss Science National Foundation (320030-163342/1), the Swiss Cancer League (KFS-3932-08-2016-R and KFS-5243-02-2021) and the Geneva Cancer League (subvention 1813).

Competing interests: The authors declare that they have no conflicts of interest.

Ethics approval: The study protocol has been approved by the Geneva Ethics Committee (Commission cantonale de la recherche scientifique de Genève [CCER], N° 2016-00141). During the study period, Geneva Cancer Registry hold all the necessary permissions to register the prognostic factors without written consent of cancer patients. Patients were informed that their data were recorded by the GCR and had the right to be ruled out from the database.

Patient consent for publication: Not required.

Data availability statement: Researchers may request access to data by contacting the corresponding author.

For peer review only

References

1. Ferlay J, Ervik M, Lam F, et al. Global Cancer Observatory: Cancer Today. Lyon, France: International Agency for Research on Cancer. Available from: https://gco.iarc.fr/today [accessed 13 April, 2022].
2. Bishehsari F, Mahdavinia M, Vacca M, *et al*. Epidemiological transition of colorectal cancer in developing countries: environmental factors, molecular pathways, and opportunities for prevention. *World J Gastroenterol* 2014;20:6055-72.
3. Stoffel EM, Mangu PB, Gruber SB, et al. Hamilton SR, Kalady MF, Lau MW, et al. Hereditary colorectal cancer syndromes: American Society of clinical oncology clinical practice guideline endorsement of the familial risk-colorectal cancer: European society for medical oncology clinical practice guidelines. *J Clin Oncol* 2015;33:209-17.
4. Marra G, Boland CR. Hereditary nonpolyposis colorectal cancer: the syndrome, the genes, and historical perspectives. *J Natl Cancer Inst* 1995;87:1114-25.
5. Fuchs CS, Giovannucci EL, Colditz GA, *et al*. A prospective study of family history and the risk of colorectal cancer. *N Engl J Med* 1994;331:1669-74.
6. Johns LE, Houlston RS. A systematic review and meta-analysis of familial colorectal cancer risk. *Am J Gastroenterol* 2001;96:2992-3003.
7. Slattery ML, Kerber RA. Family history of cancer and colon cancer risk: the Utah Population Database. *J Natl Cancer Inst* 1994;86:1618-26.
8. Antelo M, Castells A. Family history of colorectal cancer: a new survival predictor of colon cancer? *Gastroenterology* 2009;136:357-9.
9. Butterworth AS, Higgins JP, Pharoah P. Relative and absolute risk of colorectal cancer for individuals with a family history: a meta-analysis. *Eur J Cancer* 2006;42:216-27.
10. Kerber RA, Slattery ML, Potter JD, *et al*. Risk of colon cancer associated with a family history of cancer or colorectal polyps: the diet, activity, and reproduction in colon cancer study. *Int J Cancer* 1998;78:157-60.
11. Aste H, Picasso M, Allegretti A, *et al*. Correlation between family history of colorectal cancer and pathological and clinical features of the disease. *Cancer Detect Prev* 1994;18:343-8.
12. Cannon-Albright LA, Thomas TC, Bishop DT, *et al*. Characteristics of familial colon cancer in a large population data base. *Cancer* 1989;64:1971-5.
13. No authors. Clinical and pathological analyses of patients with a family history of colorectal cancer. Registry Committee, Japanese Research Society for Cancer of the Colon and Rectum. *Jpn J Clin Oncol* 1993;23:342-9.
14. Slattery ML, Kerber RA. The impact of family history of colon cancer on survival after diagnosis with colon cancer. *Int J Epidemiol* 1995;24:888-96.
15. Chan JA, Meyerhardt JA, Niedzwiecki D, *et al*. Association of family history with cancer recurrence and survival among patients with stage III colon cancer. *JAMA* 2008;299:2515-23.
16. Kao PS, Lin JK, Wang HS, *et al*. The impact of family history on the outcome of patients with colorectal cancer in a veterans' hospital. *Int J Colorectal Dis* 2009;24:1249-54.
17. Zell JA, Honda J, Ziogas A, *et al*. Survival after colorectal cancer diagnosis is associated with colorectal cancer family history. *Cancer Epidemiol Biomarkers Prev* 2008;17:3134-40.
18. Bass AJ, Meyerhardt JA, Chan JA, *et al*. Family history and survival after colorectal cancer diagnosis. *Cancer* 2008;112:1222-9.
19. Kune GA, Kune S, Watson LF. The effect of family history of cancer, religion, parity and migrant status on survival in colorectal cancer. The Melbourne Colorectal Cancer Study. *Eur J Cancer* 1992;28A:1484-7.
20. Kirchhoff AC, Newcomb PA, Trentham-Dietz A, *et al*. Family history and colorectal cancer survival in women. *Fam Cancer* 2008;7:287-92.

For peer review only - http://bmjopen.bmj.com/site/about/guidelines.xhtml

21. Bruni D, Angell HK, Galon J. The immune contexture and Immunoscore in cancer prognosis and therapeutic efficacy. *Nature Rev Cancer* 2020;20:662-80.
22. Merok MA, Ahlquist T, Røyrvik EC, *et al*. Microsatellite instability has a positive prognostic impact on stage II colorectal cancer after complete resection: results from a large, consecutive Norwegian series. *Ann Oncol* 2013;24:1274-82.
23. Seppälä TT, Böhm JP, Friman M, *et al*. Combination of microsatellite instability and BRAF mutation status for subtyping colorectal cancer. *Br J Cancer* 2015;112:1966-75.
24. Samowitz WS, Curtin K, Ma KN, *et al*. Microsatellite instability in sporadic colon cancer is associated with an improved prognosis at the population level. *Cancer Epidemiol Biomarkers Prev* 2001;10:917-23.
25. Pagès F, Mlecnik B, Marliot F, Bindea G, Ou F-S, Bifulco C, et al. International validation of the consensus Immunoscore for the classification of colon cancer: a prognostic and accuracy study. *Lancet* 26 2018;391:2128-39.

For peer review only

Figure titles

Figure 1. Sources of data and links between the datasets

Figure 2. Specific survival rates for colon cancer and rectal cancer

Figure 3. Specific survival rates for colon cancer according to stages

Figure 4. Specific survival rates for rectal cancer according to stages

For peer review only

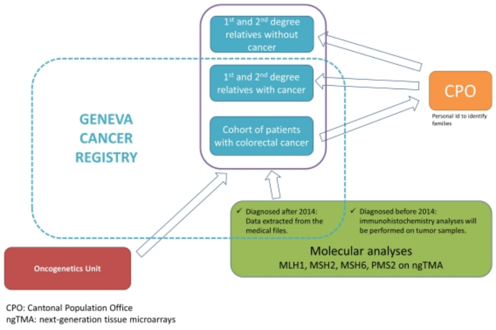

Sources of data and links between the data sets

1422x1066mm (96 x 96 DPI)

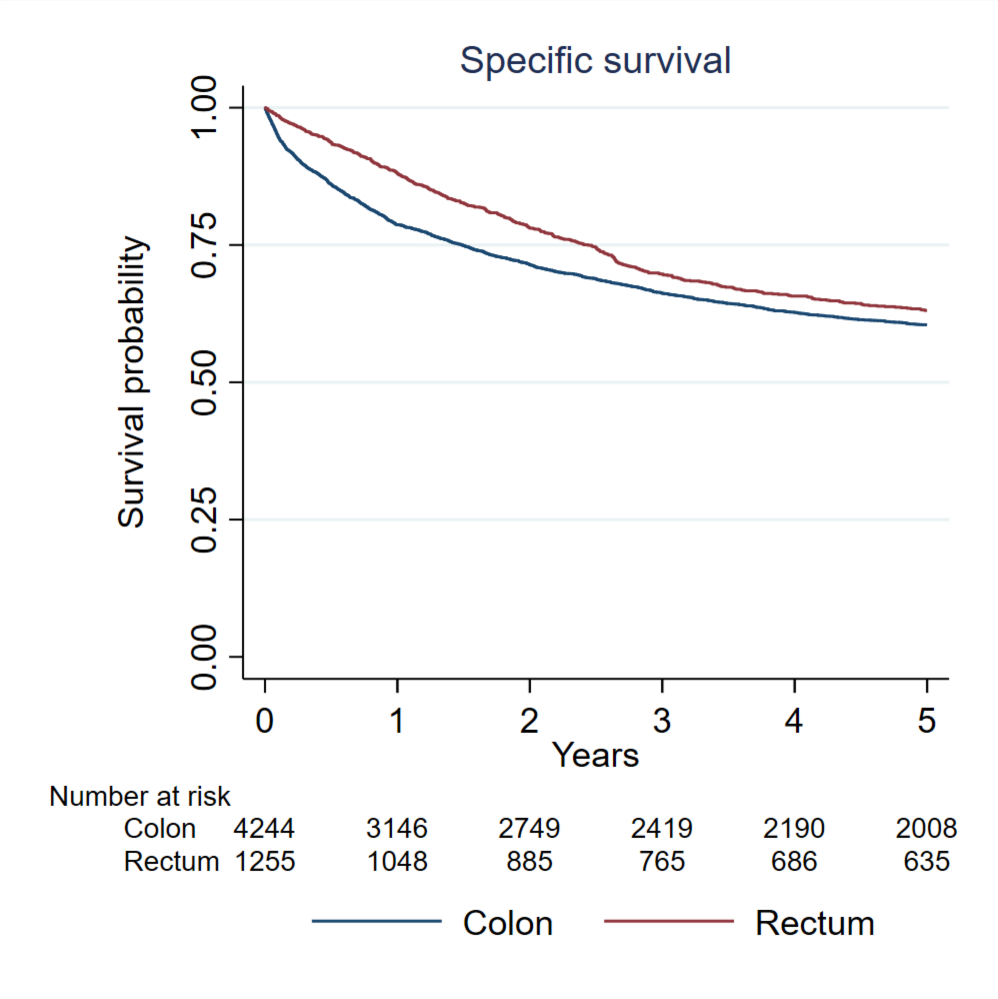

Specific survival rates for colon cancer and rectal cancer

84x84mm (300 x 300 DPI)

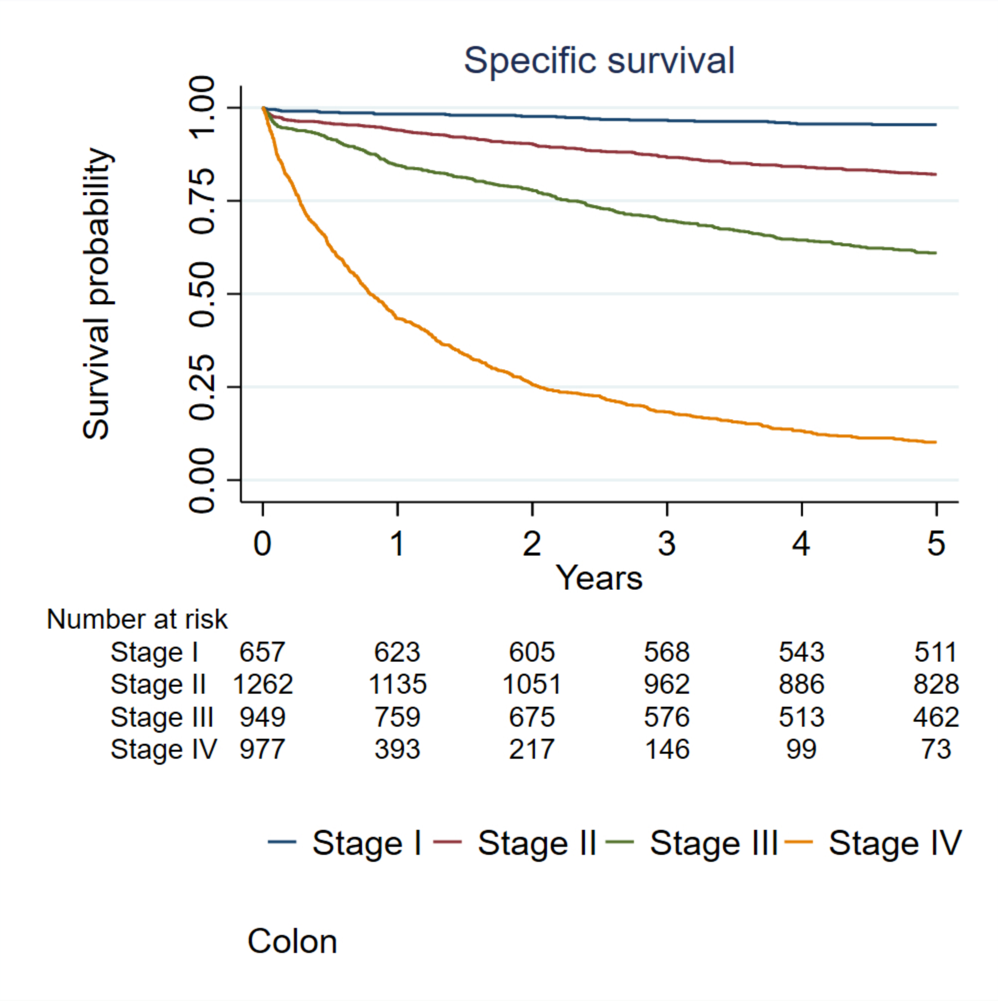

Specific survival rates for colon cancer according to stages

84x84mm (300 x 300 DPI)

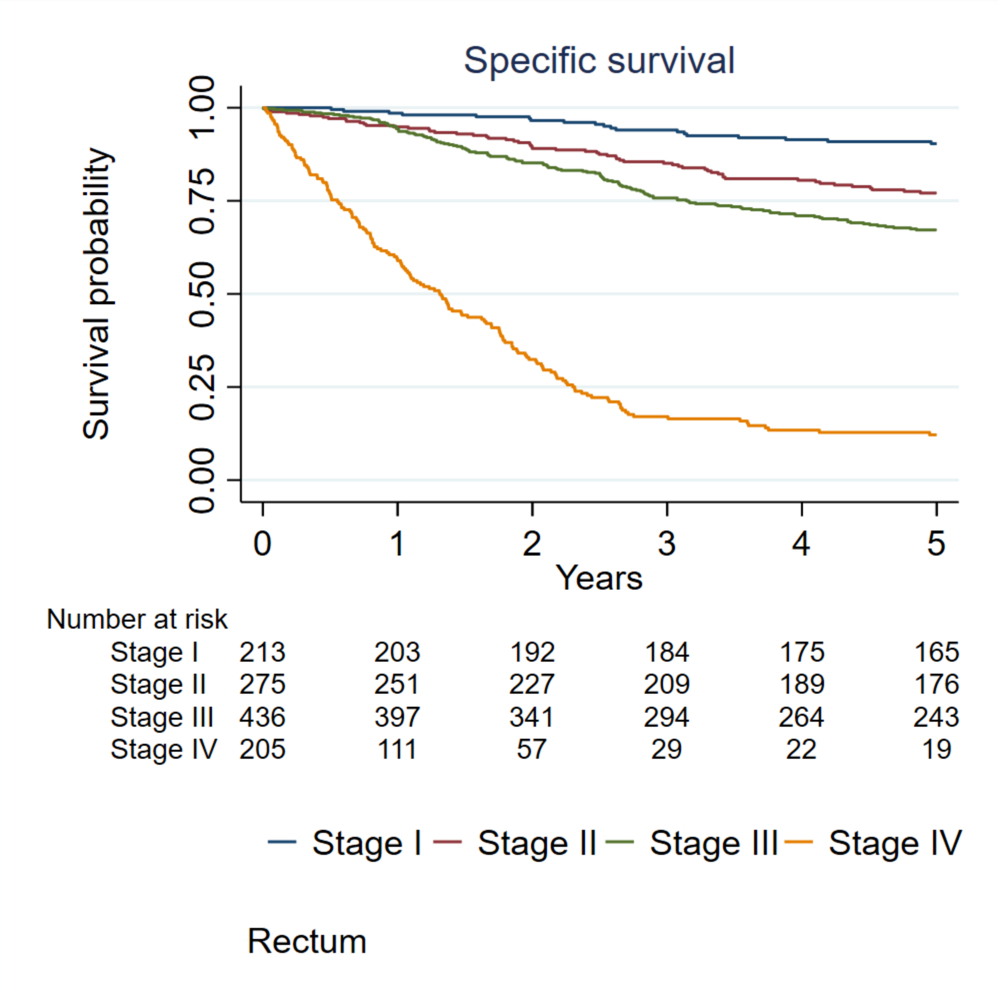

Specific survival rates for rectal cancer according to stages

84x84mm (300 x 300 DPI)

STROBE Statement—Checklist of items that should be included in reports of ***cohort studies***

	Item No	Recommendation
**Title and abstract**	1	(*a*) Indicate the study's design with a commonly used term in the title or the abstract<br>YES
		(*b*) Provide in the abstract an informative and balanced summary of what was done and what was found<br>YES
**Introduction**		
Background/rationale	2	Explain the scientific background and rationale for the investigation being reported<br>YES
Objectives	3	State specific objectives, including any prespecified hypotheses<br>YES
**Methods**		
Study design	4	Present key elements of study design early in the paper<br>YES
Setting	5	Describe the setting, locations, and relevant dates, including periods of recruitment, exposure, follow-up, and data collection<br>YES
Participants	6	(*a*) Give the eligibility criteria, and the sources and methods of selection of participants. Describe methods of follow-up<br>YES
		(*b*) For matched studies, give matching criteria and number of exposed and unexposed<br>N/A
Variables	7	Clearly define all outcomes, exposures, predictors, potential confounders, and effect modifiers. Give diagnostic criteria, if applicable<br>YES for outcomes and N/A for other variables
Data sources/ measurement	8*	For each variable of interest, give sources of data and details of methods of assessment (measurement).<br>YES<br>Describe comparability of assessment methods if there is more than one group<br>N/A
Bias	9	Describe any efforts to address potential sources of bias<br>N/A
Study size	10	Explain how the study size was arrived at<br>N/A
Quantitative variables	11	Explain how quantitative variables were handled in the analyses. If applicable, describe which groupings were chosen and why<br>N/A
Statistical methods	12	(*a*) Describe all statistical methods, including those used to control for confounding<br>N/A
		(*b*) Describe any methods used to examine subgroups and interactions<br>N/A
		(*c*) Explain how missing data were addressed<br>N/A
		(*d*) If applicable, explain how loss to follow-up was addressed<br>YES
		(*e*) Describe any sensitivity analyses

For peer review only - http://bmjopen.bmj.com/site/about/guidelines.xhtml

		N/A
**Results**		
Participants	13*	(a) Report numbers of individuals at each stage of study—eg numbers potentially eligible, examined for eligibility, confirmed eligible, included in the study, completing follow-up, and analysed<br>YES
		(b) Give reasons for non-participation at each stage<br>YES
		(c) Consider use of a flow diagram<br>NO
Descriptive data	14*	(a) Give characteristics of study participants (eg demographic, clinical, social) and information on exposures and potential confounders<br>YES
		(b) Indicate number of participants with missing data for each variable of interest<br>YES
		(c) Summarise follow-up time (eg, average and total amount)<br>YES
Outcome data	15*	Report numbers of outcome events or summary measures over time<br>YES
Main results	16	(*a*) Give unadjusted estimates and, if applicable, confounder-adjusted estimates and their precision (eg, 95% confidence interval). Make clear which confounders were adjusted for and why they were included<br>N/A
		(*b*) Report category boundaries when continuous variables were categorized<br>YES
		(*c*) If relevant, consider translating estimates of relative risk into absolute risk for a meaningful time period<br>N/A
Other analyses	17	Report other analyses done—eg analyses of subgroups and interactions, and sensitivity analyses<br>NO
**Discussion**		
Key results	18	Summarise key results with reference to study objectives<br>YES
Limitations	19	Discuss limitations of the study, taking into account sources of potential bias or imprecision.<br>YES<br>Discuss both direction and magnitude of any potential bias<br>N/A
Interpretation	20	Give a cautious overall interpretation of results considering objectives, limitations, multiplicity of analyses, results from similar studies, and other relevant evidence<br>N/A
Generalisability	21	Discuss the generalisability (external validity) of the study results<br>N/A
**Other information**		
Funding	22	Give the source of funding and the role of the funders for the present study and, if applicable, for the original study on which the present article is based<br>YES

For peer review only - http://bmjopen.bmj.com/site/about/guidelines.xhtml

For peer review only

*Give information separately for exposed and unexposed groups.

Note: An Explanation and Elaboration article discusses each checklist item and gives methodological background and published examples of transparent reporting. The STROBE checklist is best used in conjunction with this article (freely available on the Web sites of PLoS Medicine at http://www.plosmedicine.org/, Annals of Internal Medicine at http://www.annals.org/, and Epidemiology at http://www.epidem.com/). Information on the STROBE Initiative is available at http://www.strobe-statement.org.

For peer review only

For peer review only - http://bmjopen.bmj.com/site/about/guidelines.xhtml
